# The consequences of implementing non-invasive prenatal testing with cell-free foetal DNA for the detection of Down syndrome in the Spanish National Health Service: a cost-effectiveness analysis

**DOI:** 10.1186/s12962-019-0173-8

**Published:** 2019-03-01

**Authors:** J. C. Bayón, E. Orruño, M. I. Portillo, J. Asua

**Affiliations:** 1 Basque Office for Health Technology Assessment (OSTEBA), Ministry of Health, Basque Government, c/Donostia 1, 01010 Vitoria-Gasteiz, Basque Country Spain; 2 Bioaraba Health Research Institute, Methodology and Statistics Unit, Araba University Hospital, Txagorritxu Headquarters, 4th Floor, c/José Achótegui, 01009 Vitoria-Gasteiz, Basque Country Spain; 3 Colorectal and Prenatal Screening Coordinating Centre, Basque Health Service, Bilbao, Basque Country Spain

**Keywords:** Cost-effectiveness analysis, NIPT, non-invasive prenatal testing, cffDNA, Down syndrome, Trisomy 21, Prenatal screening

## Abstract

**Background:**

DNA-based non-invasive prenatal testing (NIPT) using maternal blood constitutes an emerging technology for the detection of Down syndrome (DS). The aim of the study was to conduct a cost-effectiveness analysis to evaluate the economic costs and health implications of the introduction of NIPT based on cell-free foetal DNA analysis through different screening strategies for the detection of DS.

**Methods:**

An analytical short-term decision model was developed, from the payer´s perspective (Spanish National Health Service). The main outcome measure was the number of DS cases detected. Secondary measures included associated miscarriages, women undergoing current screening, women undergoing NIPT, positive NIPT and invasive procedures performed. The study setting was the Spanish National Health Service. Three strategies were compared: (a) first- and second-trimester screening (current screening); (b) NIPT as contingent testing; and (c) NIPT as first-line testing. Modelling was based on a hypothetical cohort of 100,000 Spanish pregnant women. Population data were obtained from the database of the Basque Antenatal Screening Programme. Deterministic sensitivity analyses were performed to assess variations in the cost of NIPT, screening risk cut-off, screening uptake-rate and rate of failure of NIPT.

**Results:**

NIPT as contingent testing (strategy b) led to fewer miscarriages following invasive procedures and a slight reduction in the number of DS cases detected compared to current screening. However, lowering the screening cut-off to ≥ 1:500 would improve the overall effectiveness of NIPT as contingent testing, increasing the number of DS cases detected and decreasing foetal losses as compared to the current screening, despite there would be an extra-cost of 3.5%. When NIPT was used as first-line testing (strategy c), the screening would be more effective but also more expensive, with incremental cost-effectiveness ratios (ICERs) per additional case of DS detected of €1,299,763 and €1,232,763, compared with strategies a and b, respectively. Results were sensitive to the different parameters considered in the analysis.

**Conclusions:**

Both, as first-line testing and as contingent testing when screening cut-off was lowered ≥ 1:500, NIPT would lead to more favourable outcomes as compared to the current screening (both in terms of DS cases detected and miscarriages avoided), but at a greater cost.

## Background

Prenatal detection of Down syndrome (DS) through biochemical tests and ultrasonography has resulted in a marked decrease in births of babies with the condition and an increase in the number of elective abortions, except in countries where this option is not legally allowed [1]. In Spain, in 2005 the Spanish Society of Gynaecology and Obstetrics (SEGO), after a reflection process, proposed rolling out the first-trimester combined screening test across the Spanish National Health Service [2]. Currently, SEGO is undertaking an evaluation of the use of the aforementioned test nationwide.

In the Basque Country, based on the results of a health technology assessment report conducted by the Basque Office for Health Technology Assessment (OSTEBA) [3], the Ministry of Health approved the launch of the first-trimester screening in 2008, which consists of a combined screening test for all pregnant women receiving antenatal care within the Basque Public Health Service. The screening was piloted in 2009 and fully rolled out in 2010. Although the detection rates using the combined test are close to 90% [4], non-invasive tests based on genome sequencing and bioinformatics are newly being introduced into clinical practice. In particular, cell-free foetal DNA (cffDNA) can be detected in maternal blood between weeks 11 and 22 of pregnancy and can be used as non-invasive prenatal testing (NIPT) for DS [5]. Such tests yield similar detection rates to invasive tests (IT) based on cytogenetic analyses [6] and are revolutionising prenatal screening.

The use of NIPT has grown rapidly, leading to a simultaneous reduction in the application of first-trimester combined tests and IT [7, 8]. Recent studies indicate that NIPT can achieve an aneuploidy detection rate of 99.2% (95% confidence interval (CI) 98.5% to 99.6%), higher than that obtained with conventional serological tests, and a false positive rate of around 0.09% (95% CI 0.05% to 0.14%) for DS [9], as well as high levels of sensitivity (99.3%; 95% CI 98.9% to 99.6%) and specificity (99.9%; 95% CI 99.9% to 100%) for this chromosome anomaly [10]. Nevertheless, it should be underlined that cffDNA tests are not diagnostic, and therefore, when NIPT results are positive, the diagnosis requires confirmation by means of genetic analysis of samples collected invasively [11].

**Fig. 1:**
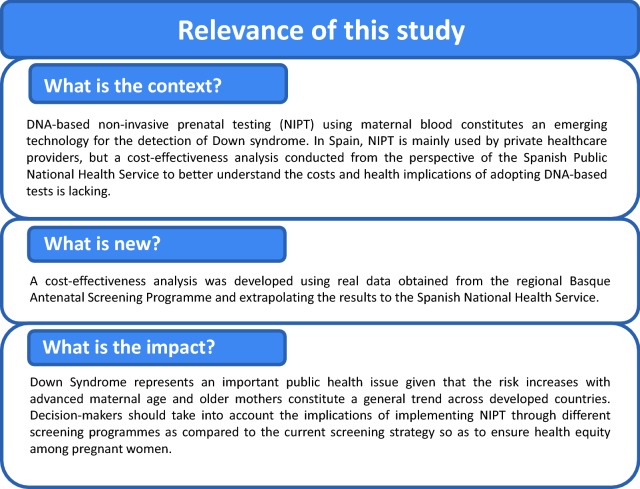
Relevance of this study

The introduction of NIPT may entail important changes in the screening strategies for DS applied within health services, and hence, there is a pressing need to assess the benefits, risks and costs of cffDNA tests (Fig. 1). From an economic view point, differences in cost-effectiveness between available techniques are not clear given the high costs of NIPT ($500 to $2100 per test in the USA [12] or €550 in our setting), despite aneuploidy detection rates are higher with NIPT than with conventional serological tests. Several studies have indicated that NIPT is cost-effective as first-line testing for DS, compared to other screening alternatives, when the analysis is performed from a societal perspective, though this is not the case when the analysis is conducted from the payer´s perspective [13]. A recent study indicates that the use of NIPT for first-line testing is beneficial in terms of the number of cases of DS detected and the reduction in the number of miscarriages following IT, although with significantly higher associated costs than current (first and second trimester) screening [14]. When NIPT is used for contingent testing in high-risk pregnancies during the first or second trimester of gestation, lower associated costs and fewer foetal losses have been observed [14–16]. Nevertheless, unlike first- and second-trimester screening tests, this approach would not provide early identification of other foetal abnormalities and pregnancies at risk of preeclampsia [17] or intrauterine growth restriction [18].

In this context, the aim of this study was to carry out a cost-effectiveness analysis to evaluate the economic costs and health implications of the introduction of NIPT through different screening strategies for the detection of DS, as compared to the current combined screening test.

## Methods

### Analytical decision model

An analytical decision model was developed in Excel to evaluate the costs (euros of 2015) and consequences of introducing NIPT for the detection of DS. The analysis was carried out from the payer´s perspective (Spanish National Health Service), and hence only direct healthcare costs associated with screening were considered, with a short time horizon, between week 10 of pregnancy and labour. Since the model was short-term, costs or effects were not discounted.

The primary outcome measure was the number of cases of trisomy 21 (T21) detected, not taking into account miscarriages occurring between diagnosis and birth or the personal decision of whether to continue with or terminate the T21 pregnancy. Secondary measures included the number of miscarriages associated to IT, the number of women undergoing first- and second-trimester screening, the number of women undergoing NIPT, the number of NIPT with positive results and invasive procedures performed. The model considered short-term outcomes, i.e. birth and/or interruption of pregnancy, thus long-term effects related to the infant with DS were not taken into account.

### Study setting and location

The ultimate study setting was the entire Spanish National Health Service. Nevertheless, the study was conducted on the basis of pregnancy outcomes registered in the Basque Health Service at a regional level. Both, the Spanish National Health Service and the Basque Health Service (regional autonomous health service) provide comprehensive healthcare to the entire population and, thus, offer prenatal diagnosis and pregnancy surveillance for all pregnant women. No fees are required for the provision of these services.

The prenatal screening is generally carried out in Primary Care centers with the involvement of midwives in collaboration with GPs and gynecologists. Different aspects of the prenatal screening (e.g. informed consent, prenatal assessment, different procedures conducted, pregnancy outcomes (i.e. births, stillbirths and termination of pregnancies, etc.) are registered in a common medical record using specific software (Basque Antenatal Screening Programme for DS and other chromosome anomalies (ASP)).

### Comparison of different interventions

**Fig. 2:**
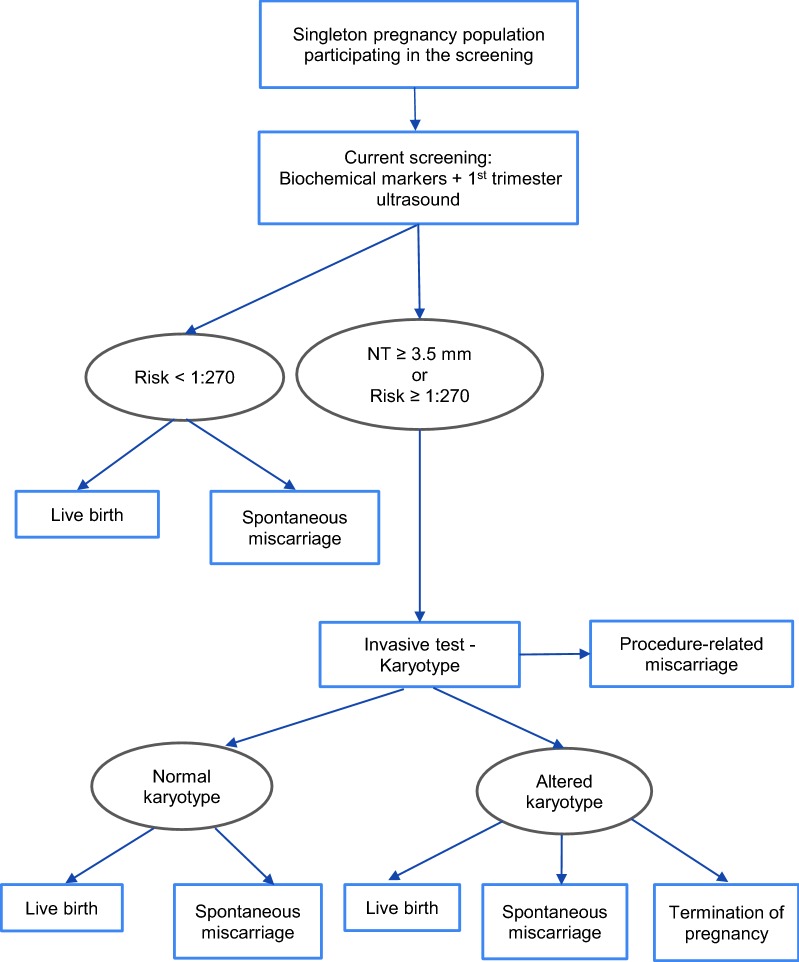
Diagram representing the first- and second-trimester screening (current screening)

**Fig. 3:**
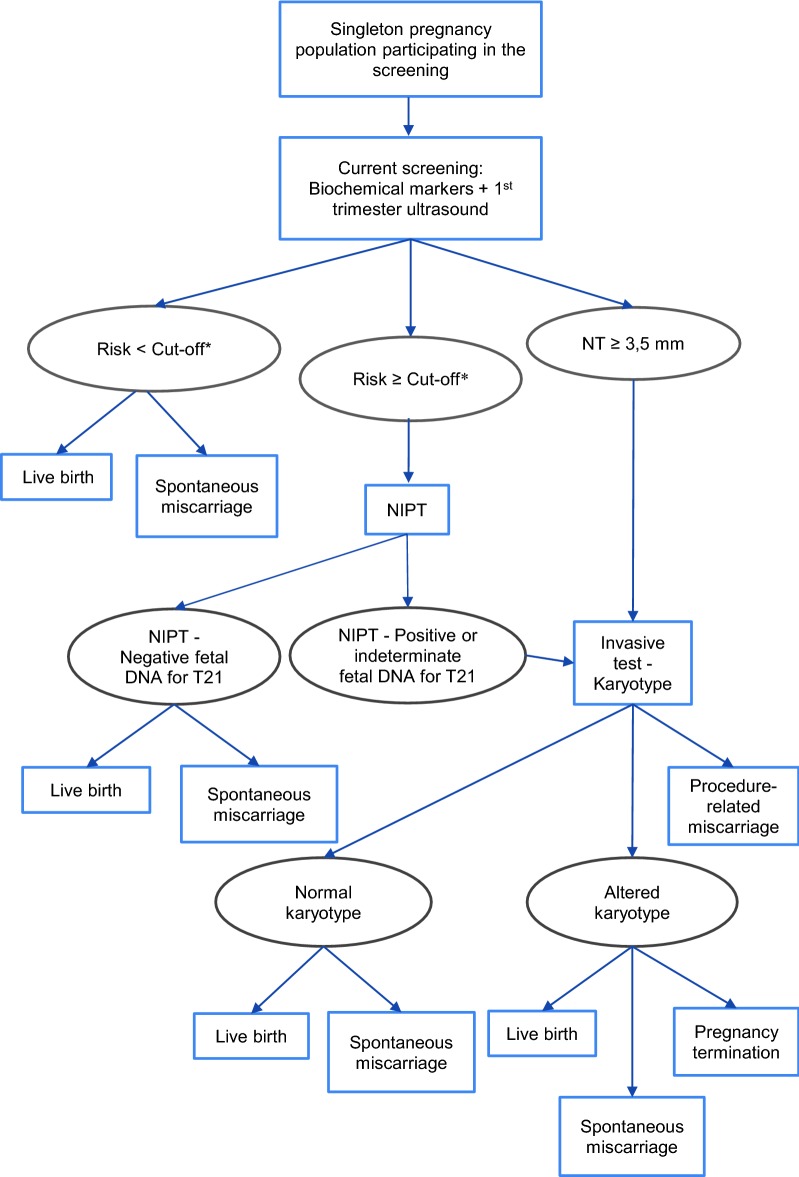
Diagram representing the use of cffDNA-based NIPT as contingent testing. *The screening cut-off for the base case was set at 1:270 and for the univariate sensitivity analysis was set at 1:500 or at 1:1000

**Fig. 4:**
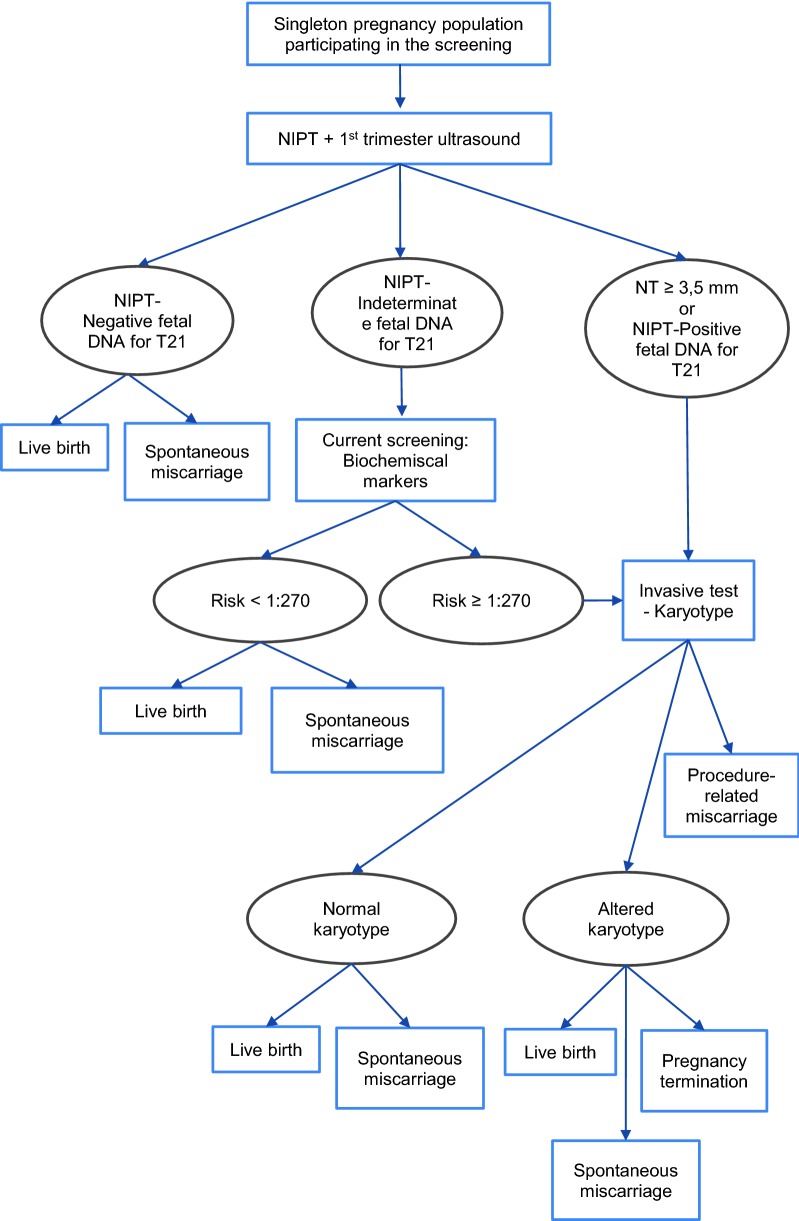
Diagram representing the use of cffDNA-based NIPT as first-line testing

The three strategies compared in the analysis were: (a) first- and second-trimester screening (current screening); (b) cffDNA-based NIPT as contingent testing (screening test); and (c) cffDNA-based NIPT as first-line testing (Figs. 2, 3 and 4).

The strategy for the first- and second-trimester prenatal screening (strategy a) consisted of a combined test using ultrasound markers [nuchal translucency (NT)] and serological markers (pregnancy-associated plasma protein-A (RAPP-A) and free β-human chorionic gonadotrophin (β-HCG) in the first trimester and free β-HCG and alpha-fetoprotein (α-FP) in the second trimester). For the risk calculation, maternal age was included and adjusted for modifying factors (body weight, ethnicity and previous history of chromosome anomalies, among others), using the Ssdwlab^®^ software; a cut-off of ≥ 1:270 was set by consensus and pregnant women classified as high-risk were offered an invasive procedure, i.e. amniocentesis or chorionic villus sampling (CVS), to obtain samples for cytogenetic analysis that would confirm the diagnosis.

Second, NIPT as contingent testing (strategy b) would be offered in high-risk pregnancies (≥ 1:270) after the first- and second-trimester prenatal screening tests. Third, NIPT as first-line testing (strategy c) would replace serological markers of first- and second-trimester screening, maintaining the first-trimester NT test, since it constitutes a key test in the prenatal screening for DS and other chromosomal anomalies. Pregnant women with no results or inconclusive results in NIPT would be offered the current screening tests (strategy a). If the results of NIPT or the first- and second-trimester serological screening markers were positive, women would be offered invasive procedures to confirm the diagnosis based on cytogenetic analysis.

### Population

To develop the analytical decision model, we started with a hypothetical cohort of 100,000 pregnant women, calculating who would participate in the first- and second-trimester screening tests for the detection of T21. In the population-based calculation, we took into account the absolute risk of miscarriage during pregnancy [19] and estimated both, the number of births and pregnancies. We excluded women who attended private clinics for antenatal care, those who although they received antenatal care through the national health service declined prenatal screening tests and those who initially agreed but did not complete the process, as well as any woman with twin pregnancies, even if one of the foetuses was lost during gestation. Finally, the model was based on the number of singleton pregnancies at week 14 of gestation.

Population data were obtained for the period 2010–2013 from the database of the Basque Antenatal Screening Programme (ASP) for DS and other chromosome anomalies and from the data on all births in the Basque Country published by the Basque Statistics Institute (EUSTAT).

### Variables included in the model (see Table 1)

#### First- and second-trimester screening

Based on the population calculations, we estimated that 78.38% of women with a singleton pregnancy underwent first- and second-trimester screening. Of these, 96.85% corresponded to first-trimester and 3.15% to second-trimester tests (source: ASP). We assumed that 33%, 34% and 33% of first-trimester biochemical tests were carried out sequentially in weeks 11, 12 and 13 of pregnancy, respectively, while the NT test was carried out in week 12.

**Table 1:** Key model inputs (base case)

Parameter	Value	Source
*Pregnant population attending the NHS for pregnancy follow*-*up*	78.40%	Estimated from EUSTAT^a^ and ASP^b^
*Twin pregnancies*	2.30%	ASP
*Pregnant population rejecting the screening*	0.01%	ASP
*Pregnant population stopping the screening*	0.02%	ASP
Prevalence of Down Syndrome	0.43%	ASP
*First and second trimester screening*		
1st and 2nd trimester screening uptake	78.38%	Estimated
1st trimester screening tests performed	96.85%	ASP
Week 11	33%	Estimated
Week 12	34%	Estimated
Week 13	33%	Estimated
2nd trimester screening tests performed	3.15%	ASP
High-risk pregnant population (NT > 3.5 mm) for T21	0.41%	ASP
Prevalence of T21	1:10	Hulstaert et al. [20]
Sensitivity	89.75%	ASP
Specificity	95.65%	ASP
*NIPT*		
NIPT as first line screening uptake	78.38%	Assumption
NIPT repeated	4%	Hulstaert et al. [20]
NIPT without results (test failure)	2%	Hulstaert et al. [20]
Sensitivity	99.30%	Benn et al. [25]
Specificity	99.84%	Benn et al. [25]
*Invasive testing (CVS or amniocentesis)*		
IT rejected	4.82%	ASP
Procedure-related fetal loss	0.69%	ASP
Hospitalization for amniotic fluid leakage	1%	Hulstaert et al. [20]
Sensitivity	100%	Assumption
Specificity	100%	Assumption
Pregnancy termination after T21 diagnosis	94%	ASP
*Miscarriage in the total pregnant population*		Ammon Avalos et al. [19]
Week 10	5%	
Week 12	2.50%	
Week 14	1.50%	
*Miscarriage in the T21 pregnant population*		Snijders et al. [28]
Week 10	36%	
Week 12	30%	
Week 14	25%	
*Costs*		
Primary care appointment with the midwife	€24	2015 Osakidetza fees^c^
Collection of the blood sample	€19	2015 Osakidetza fees
Management of the request for the blood test	€5	2015 Osakidetza fees
PAPP-A	€14	2015 Osakidetza fees
β-hCG	€14	2015 Osakidetza fees
AFP	€14	2015 Osakidetza fees
Ultrasound monitoring of amniocentesis procedure	€338	2015 Osakidetza fees
Amniotic fluid karyotyping	€451	2015 Osakidetza fees
CVS karyotyping	€840	2015 Osakidetza fees
Unit cost of NIPT	€550	Estimation
Pregnancy termination (DRG 381)	€1825	2015 Osakidetza fees
Hospitalization due to amniotic fluid leakage (DRG 886)	€1577	2015 Osakidetza fees

AFP, alpha-fetoprotein; CVS, chorionic villus sampling; DRG, diagnosis-related group; IT, invasive tests; NT, Nuchal translucency; PAPP-A, pregnancy associated plasma protein A; β-hCG, free fraction of the β subunit of the human chorionic gonadotropin; T21, trisomy 21

^a^Basque Statistics Institute (EUSTAT)

^b^Basque Antenatal Screening Programme (ASP) for Down syndrome and other chromosome anomalies

^c^2015 list of fees for invoicing healthcare and teaching services in the Basque Health Service, Osakidetza

Overall, 0.41% of pregnancies were considered to be high-risk based on measurement of nuchal fold thickness (≥ 3.5 mm), corresponding to a prevalence of general congenital malformation of 1:10 [20]; the sensitivity and specificity of first- and second-trimester screening, for the T21 screening risk cut-off of 1:270, were 89.75% (95% CI 85.95% to 93.56%) and 95.65% (95% CI 95.48% to 95.82%), respectively, and the prevalence of DS was 0.43% (source of all data: ASP).

The cost of first- and second-trimester screening tests was calculated by adding to the costs of the primary care appointment with the midwife, those associated with collection of the blood sample, with the management of the request for blood testing and with the laboratory analysis and validation of the biochemical markers (source: 2015 List of fees for invoicing healthcare and teaching services in the Basque Health Service, Osakidetza).

#### DNA-based non-invasive prenatal tests

For NIPT as first-line testing (strategy c), we assumed that, as for first- and second-trimester screening, the antenatal screening coverage was 78.38%, 0.41% of pregnancies were considered high-risk (NT ≥ 3.5 mm), corresponding to a prevalence of 1:10 of general congenital malformations and a prevalence of DS of 0.43%. Given that between 1 and 8% of NIPT fail due to insufficient foetal fraction in the samples [21–24], in the model, we assumed that 2% of NIPT would not provide valid results or that the results would be inconclusive precluding adequate interpretation.

Drawing on the scientific evidence, we assumed that the sensitivity and specificity of NIPT for T21 would be 99.3% (95% CI 98.2% to 99.8%) and 99.84% (95% CI 99.69% to 99.92%), respectively [25, 26]. The cost of the non-invasive testing was estimated to be €550 per test, based on the fees charged by some laboratories to carry out NIPT for private clinics at the time the analysis was conducted. We did not take into account the additional costs related to genetic counselling, assuming that the process would be similar to usual screening (strategy a). To that initial cost, we added the costs of second tests when the sample from the first test did not render any results, given that it is estimated that 4% of tests are repeated in week 12 of pregnancy [20].

#### Invasive procedures

Overall, 4.82% of pregnant women with positive results in the first- and second-trimester screening tests declined invasive procedures to confirm the diagnosis (source: ASP). For NIPT with positive results, we assumed the same percentage of women would decline confirmatory invasive testing. Further, among the invasive procedures, 83% were amniocentesis and 17% CVS (source: ASP). The sensitivity and specificity of invasive procedures were both assumed to be 100%.

Regarding the adverse effects associated to IT, the rate of miscarriages related to the procedures was 0.69% (source: ASP) and there was an estimated 1% risk of hospitalization for 1 week due to amniotic fluid loss caused by ruptured membranes [27]. Neither neonatal respiratory distress syndrome nor congenital pneumonia were considered in the model.

The cost of invasive procedures was obtained by summing 83% of the cost of amniotic fluid karyotyping, 17% of the cost of CVS and the costs associated with ultrasonographic monitoring. With regard to adverse effects, it was assumed that miscarriages related to the procedure did not increase the initial costs and the costs associated with a hospital stay (€1577) were calculated in line with those for the diagnosis-related group (DRG) 886 “Other antepartum diagnoses without surgery” stated in the 2015 List of fees for invoicing healthcare and teaching services in the Basque Health Service (Osakidetza).

#### Elective abortion

Overall, 94% of women underwent elective abortion (€1825) due to a diagnosis of T21 confirmed by IT (source: ASP). The costs associated with elective abortion were quantified based on the DRG 381 “Abortion with dilation and curettage, aspiration curettage or hysterectomy” stated in the aforementioned 2015 List of fees. The rate of elective abortion was modelled in a single step between weeks 14 and 40 of pregnancy.

#### Miscarriages

In the model, miscarriages were taken into account for T21 and non-T21 pregnancies. The general rates of miscarriage were 5%, 2.5% and 1.5% at weeks 10, 12 and 14 of pregnancy, respectively [19], while for T21 pregnancies the rates were 36%, 30% and 25% at weeks 10, 12 and 14, respectively [28]. Women who had miscarriages were excluded from the model, with no impact on costs or benefits.

### Economic analysis

An economic analysis was carried out to determine which of the DS screening strategies analysed was the most cost-effective. With this purpose in mind, we calculated the incremental cost-effectiveness ratio (ICER) for each strategy compared to the others. The ICER was calculated as the ratio between the incremental cost and the incremental effectiveness (ICER = ΔC/ΔE). This ratio indicates the incremental cost of the use of one screening strategy compared to another per additional case of DS diagnosed.

#### Sensitivity analysis

Univariate and bivariate sensitivity analyses were conducted to study the potential uncertainty of some variables included in the model. The parameters were selected according to the available scientific evidence and expert opinion bearing in mind the adoption of NIPT for prenatal DS screening. In the univariate analysis, the impact on outcomes of the following changes was assessed:

First, the cost per NIPT could decrease from €550 (base case) to €150 (potential price offered by private laboratories to The Basque Health Service (Osakidetza) if NIPT was adopted) or to €76 (the same price as biochemical first- and second-trimester screening tests) due to potential economies of scale related to a greater demand if NIPT was to be adopted as the primary antenatal screening tool.

Second, in accordance with the views of obstetricians and the Spanish Society of Gynaecology and Obstetrics, the screening cut-off could be set up to 1:500 or 1:1000 instead of 1:270 (base case) (Table 2), when NIPT was used as contingent testing vs. first- and second-trimester screening.

**Table 2:** Sensitivity and specificity on the basis of the screening cut-off. Source: Basque Antenatal Screening Program (ASP) for Down syndrome and other chromosome anomalies

Risk cut-off point	TP	FP	FN	TN	Sensitivity (%)	Specificity (%)	FPR (%)	FNR (%)
1:270	219	2.45	25	53.88	89.75	95.65	4.35	10.25
1:500	225	3.99	19	52.34	92.21	92.91	7.09	7.79
1:1000	230	7.19	14	49.14	94.26	87.23	12.77	5.74

FN, false negative; FNR, false-negative ratio; FP, false positive; FPR, false positive ratio; TN, true negative; TP, true positive

Third, assuming the Spanish Health Service offered NIPT as first-line testing as part of the prenatal screening, the uptake-rate could increase as a result of a transfer of women from the private sector to the National Health Service (NHS). Thus, the impact of a rise in the rate of screening uptake from 78.8 to 89.97%, as a result of an increase from 78.50 to 90.00% in the number of women receiving antenatal care in the NHS if NIPT were used as first-line antenatal testing was analysed.

Fourth, in accordance with the scientific literature, the rate of analytic failure (failure of the cffDNA testing) ranged from 0 to 12.7% [29]. Taken into consideration this variability, the impact of the variation of NIPT failure from 0 to 12% on results was examined.

In the bivariate analysis, we analysed the impact on results of a rise in the rate of screening uptake from 78.38 to 89.97%, as a result of an increase from 78.50 to 90.00% in the number of women receiving antenatal care through the Spanish National Health Service if NIPT was used as first-line antenatal testing, together with a reduction in the cost per NIPT from €550 to €150 or €76 due to potential economies of scale related to a greater demand owing to the higher coverage.

## Results

### Economic analysis

**Fig. 5:**
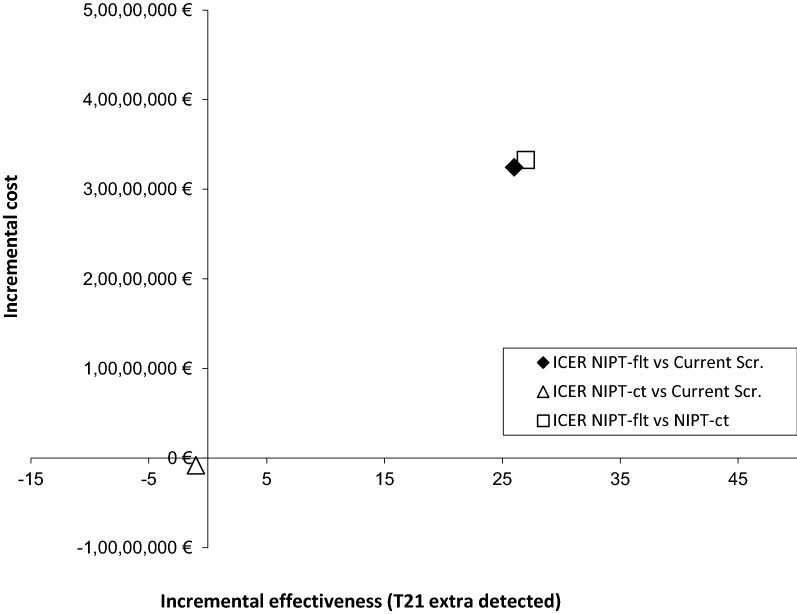
Incremental cost-effectiveness plane for the case base.* Current Scr.* Current first- and second-trimester screening,* ICER* Incremental cost-effectivenes, *NIPT-ct* DNA-based non-invasive prenatal testing as contingent testing, *NIPT-flt* DNA-based non-invasive prenatal testing as first-line testing

The results for each of antenatal DS screening strategies analysed are presented in Fig. 5 and Table 3, including the base case (strategy a), with a screening coverage of 78.38% of pregnant women in whom the screening would be performed at 14 weeks of pregnancy (corresponding to 67,074 women, with a T21 screening cut-off of ≥ 1:270 and a cost per NIPT of €550). Strategy b, in which NIPT was used as contingent testing led to fewer miscarriages following invasive procedures and a slight reduction in the number of cases of T21 detected compared to current screening. Therefore, the screening would be less costly but would also be less effective. Strategy c, in which NIPT was used as first-line testing compared to the current screening or the use of NIPT as contingent testing, would lead to a fewer miscarriages following invasive procedures and more cases of T21 being detected, but at a higher cost; that is, the screening would be more effective but also more expensive. Specifically, the ICERs per additional case of DS detected were €1,299,763 for NIPT as first-line testing as compared to current screening and €1,232,763 for NIPT as first-line testing as compared to NIPT as contingent testing, respectively.

**Table 3:** Cost-effectiveness analysis results (base case)

Screening strategy	1st and 2nd trimester screening (current screening)	NIPT as contingent testing	NIPT as first-line testing
*Effectiveness*			
No of women undergoing 1st and 2nd trimester screening tests	66,799	66,799	1336
No of women undergoing NIPT	0	3152	66,799
No of NIPT with a positive result	0	251	280
No of IT^a^	3275	579	700
No of procedure-related miscarriages	23	4	5
No of T21 cases detected	271	269	296
*Costs*			
1st and 2nd trimester screening tests	€5,292,716	€5,292,716	€101,536
NIPT	€0	€1,802,350	€40,114,800
IT	€3,093,565	€546,923	€661,220
Hospitalisation due to amniotic fluid leakage and pregnancy termination owing to T21	€515,591	€469,362	€518,389
Total costs	€8,901,872	€8,111,351	€41,395,745

Economic analysis	NIPT as first-line testing vs 1st and 2nd trimester screening	NIPT as contingent testing vs 1st and 2nd trimester screening	NIPT as first-line testing vs NIPT as contingent testing
Incremental cost	€32,494,073	€− 790,521	€33,284,594
Incremental effectiveness (T21 extra cases detected)	25	− 2	27
ICER (€/T21 extra case detected)	1,299,763	–	1,232,763

ICER, incremental cost-effectiveness ratio; IT, invasive tests; NIPT, non-invasive prenatal testing; T21, trisomy 21

^a^Out of the total number of invasive diagnostic tests performed, 271 correspond to pregnant women with a NT ≥ 3.5 mm (considered high-risk)

The results of the sensitivity analysis are reported in Tables 4, 5, 6, 7 and 8. At a cost of €150 per NIPT, the strategy that used NIPT as first-line testing (strategy c) would be both, more effective and more expensive than the current screening (strategy a) or to the use of NIPT as contingent testing (strategy b) (ICER of €132,787 and of €200,787 per additional case of DS detected, respectively). At a cost of €76 per NIPT, strategy c would be more effective and less expensive than strategy a, which would make it the dominant strategy and more effective but more expensive than strategy b (ICER of €9860 per additional case of DS detected).

**Table 4:** Univariate sensitivity analysis: when the cost per NIPT is €150 or €76

Screening strategy	1st and 2nd trimester screening (current screening)	NIPT as contingent testing	NIPT as first-line testing
*Effectiveness*			
No of women undergoing 1st and 2nd trimester screening tests	66,799	66,799	1336
No of women undergoing NIPT	0	3152	66,799
No of NIPT with a positive result	0	251	280
No of IT^a^	3275	579	700
No of procedure-related miscarriages	23	4	5
No of T21 cases detected	271	269	296
*Costs*			
1st and 2nd trimester screening tests	€5,292,716	€5,292,716	€101,536
NIPT = €150	€0	€491,550	€10,940,400
NIPT = €76	€0	€249,052	€5,543,136
IT	€3,093,565	€546,923	€661,220
Hospitalisation due to amniotic fluid leakage and pregnancy termination owing to T21	€515,591	€469,362	€518,389
Total costs when NIPT = €150	€8,901,872	€6,800,551	€12,221,545
Total costs when NIPT = €76	€8,901,872	€6,558,053	€6,824,281

Economic analysis	NIPT as first-line testing vs 1st and 2nd trimester screening	NIPT as contingent testing vs 1st and 2nd trimester screening	NIPT as first-line testing vs NIPT as contingent testing
Incremental cost when TPNI = €150	€3,319,673	€− 2,101,321	€5,420,994
Incremental cost when TPNI = €76	€− 2,077,591	€− 2,343,819	€266,228
Incremental effectiveness (T21 extra cases detected)	25	− 2	27
ICER (€/case T21 extra detected) when NIPT = €150	132,787	–	200,777
ICER (€/case T21 extra detected) when NIPT = €76	Dominant	–	9860

ICER, incremental cost-effectiveness ratio; IT, invasive tests, NIPT: non-invasive prenatal testing; T21, trisomy 21

^a^Out of the total of the total number of invasive diagnostic tests performed, 271 correspond to pregnant women with a NT ≥ 3.5 mm (considered high-risk)

**Table 5:** Univariate sensitivity analysis: when the screening risk cut-off is set to 1:500 or 1:1000

Testing strategy	1st and 2nd trimester screening (current screening)	NIPT as contingent testing	
		Risk 1:500	Risk 1:1000
*Effectiveness*			
No of women undergoing 1st and 2nd trimester screening tests	66,799	66,799	66,799
No of women undergoing NIPT	0	4979	8763
No of NIPT with a positive result	0	259	264
No of IT^a^	3275	623	705
No of procedure-related miscarriages	23	4	5
No of T21 cases detected	271	276	281
*Costs*			
1st and 2nd trimester screening tests	€5,292,716	€5,292,716	€5,292,716
NIPT	€0	€2,847,350	€5,011,600
IT	€3,093,565	€588,486	€665,943
Hospitalisation due to amniotic fluid leakage and pregnancy termination owing to T21	€515,591	€482,137	€492,839
Total costs	€8,901,872	€9,210,689	€11,463,098

Economic analysis	NIPT as first-line testing vs 1st and 2nd trimester screening	NIPT as contingent testing vs 1st and 2nd trimester screening	
		Risk 1:500	Risk 1:1000
Incremental cost	€32,494,073	€308,817	€2,561,226
Incremental effectiveness (T21 extra cases detected)	25	5	10
ICER (€/T21 extra case detected)	1,299,763	61,763	256,123

ICER, incremental cost-effectiveness ratio; IT, invasive tests; NIPT, non-invasive prenatal testing; T21, trisomy 21

^a^Out of the total number of invasive diagnostic test performed, 271 correspond to pregnant women with a NT ≥ 3.5 mm (considered high-risk)

**Table 6:** Univariate sensitivity analysis: when the screening uptake increases from 78.8 to 89.97%

Testing strategy	1st and 2nd trimester screening (current screening)	NIPT as contingent testing	NIPT as first-line testing
			Screening uptake of 89.97%
*Effectiveness*			
No of women undergoing 1st and 2nd trimester screening tests	66,799	66,799	1534
No of women undergoing NIPT	0	3152	76,684
No of NIPT with a positive result	0	251	322
No of IT^a^	3275	579	805
No of procedure-related miscarriages	23	4	5
No of T21 cases detected	271	269	341
*Costs*			
1st and 2nd trimester screening tests	€5,292,716	€5,292,716	€116,584
NIPT	€0	€1,802,350	€46,050,950
IT	€3,093,565	€546,923	€760,403
Hospitalization due to amniotic fluid leakage and pregnancy termination owing to T21	€515,591	€469,362	€596,616
Total costs	€8,901,872	€8,111,351	€47,524,553

Economic analysis	NIPT as first-line testing vs 1st and 2nd trimester screening	NIPT as contingent testing vs 1st and 2nd trimester screening	NIPT as first-line testing vs NIPT as contingent testing
Incremental cost	€38,622,681	€− 790,521	€39,413,202
Incremental effectiveness (T21 extra cases detected)	70	− 2	72
ICER (€/T21 extra case detected)	551,753	–	547,406

ICER, incremental cost-effectiveness ratio; IT, invasive tests; NIPT, non-invasive prenatal testing; T21, trisomy 21

^a^Out of the total number of invasive diagnostic test performed, 271 correspond to pregnant women with a NT ≥ 3.5 mm (considered high-risk)

**Table 7:** Univariate sensitivity analysis: when the rate of analytic failure of NIPT varies from 0 to 12%

Testing strategy	1st and 2nd trimester screening (current screening)	NIPT as contingent testing		NIPT as first-line testing	
		Failure rate of 0%	Failure rate of 12%	Failure rate of 0%	Failure rate of 12%
*Effectiveness*					
No of women undergoing 1st and 2nd trimester screening tests	66,799	66,799	66,799	0	8016
No of women undergoing NIPT	0	3152	3152	66,799	66,799
No of NIPT with a positive result	0	256	225	286	252
No of IT^a^	3275	523	853	648	964
No of procedure-related miscarriages	23	4	6	5	7
No of T21 cases detected	271	269	269	297	294
*Costs*					
1st and 2nd trimester screening tests	€5,292,716	€5,292,716	€5,292,716	0€	€609,216
NIPT	€0	€1,733,600	€1,940,400	38,608,900€	€43,126,050
IT	€3,093,565	€494,026	€805,744	612,101€	€910,594
Hospitalisation due to amniotic fluid leakage and pregnancy termination owing to T21	€515,591	€467,785	€474,093	520,214€	€519,470
Total costs	€8,901,872	€7,988,127	€8,512,953	39,741,215€	€45,165,330

Economic analysis	NIPT as first-line testing vs 1st and 2nd trimester screening		NIPT as contingent testing vs 1st and 2nd trimester screening		NIPT as first-line testing vs NIPT as contingent testing	
	Failure rate of 0%	Failure rate of 12%	Failure rate of 0%	Failure rate of 12%	Failure rate of 0%	Failure rate of 12%
Incremental cost	€30,839,343	36,263,458	€-913,745	€-388,919	€31,753,688	€36,652,378
Incremental effectiveness (T21 extra cases detected)	26	23	− 2	− 2	28	25
ICER (€/T21 extra case detected)	1,186,129	1,576,672	–	–	1,134,039	1,466,095

ICER, incremental cost-effectiveness ratio; IT, invasive tests; NIPT, non-invasive prenatal testing; T21, trisomy 21

^a^Out of the total number of invasive diagnostic test performed, 271 correspond to pregnant women with a NT ≥ 3.5 mm (considered high-risk)

**Table 8:** Bivariate sensitivity analysis

Testing strategy	1st and 2nd trimester screening (current screening)	NIPT as contingent testing	NIPT as first-line testing
			Screening uptake of 89.97%
*Effectiveness*			
No of women undergoing 1st and 2nd trimester screening tests	66,799	66,799	1534
No of women undergoing NIPT	0	3152	76,684
No of NIPT with a positive result	0	251	322
No of IT^a^	3275	579	805
No of procedure-related miscarriages	23	4	5
No of T21 cases detected	271	269	341
*Costs*			
1st and 2nd trimester screening tests	€5,292,716	€5,292,716	€116,584
NIPT = €150	€0	€491,550	€12,559,350
NIPT = €76	€0	€249,052	€6,363,404
IT	€3,093,565	€546,923	€760,403
Hospitalisation due to amniotic fluid leakage and pregnancy termination owing to T21	€515,591	€469,362	€596,616
Total cost when NIPT = €150	€8,901,872	€6,800,551	€14,032,953
Total cost when NIPT = €76	€8,901,872	€6,558,053	€7,837,007

Economic analysis	NIPT as first-line testing vs 1st and 2nd trimester screening	NIPT as contingent testing vs 1st and 2nd trimester screening	NIPT as first-line testing vs NIPT as contingent testing
Incremental cost when TPNI = €150	€5,131,081	€− 2,101,321	€7,232,402
Incremental cost when TPNI = €76	€− 1,064,865	€− 2,343,819	€1,278,954
Incremental effectiveness (T21 extra cases detected)	70	− 2	72
ICER (€/T21 extra case detected) when NIPT = €150	73,301	–	100,450
ICER (€/T21 extra case detected) when NIPT = €76	Dominant	–	17,763

ICER, incremental cost-effectiveness ratio; IT, invasive tests; NIPT, non-invasive prenatal testing; T21, trisomy 21

^a^Out of the total number of invasive diagnostic test performed, 271 correspond to pregnant women with a NT ≥ 3.5 mm (considered high-risk)

**Fig. 6:**
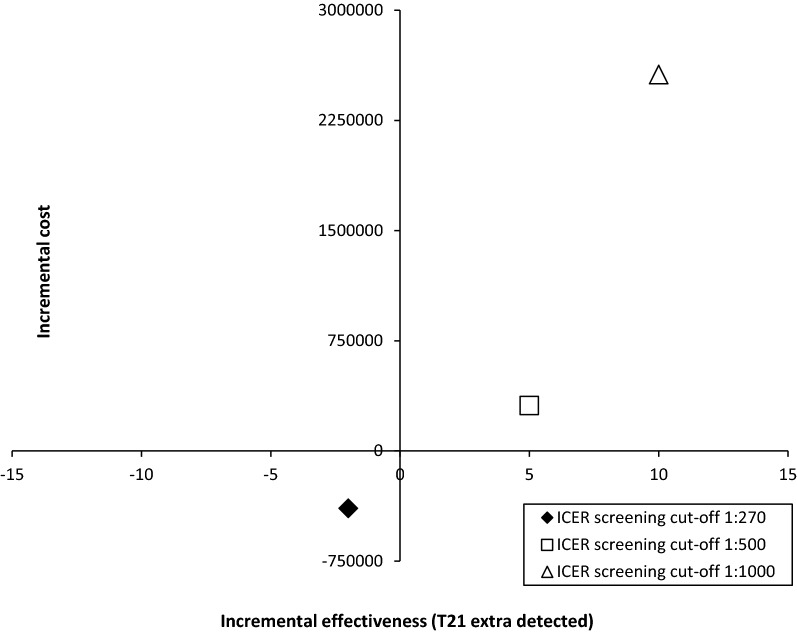
Incremental cost-effectiveness plane for the univariate sensitivity analysis with varying screening cut-offs. NIPT as contingent testing versus current first- and second-trimester screening.* ICER* Incremental cost-effectivenes

If the screening risk cut-off was modified to 1:500 or to 1:1000, strategy b would be more effective in terms of the number of DS cases detected (276 and 281 DS cases detected as compared to 271 with the current first- and second-trimester screening) and in terms of the number of IT related miscarriages (decreasing from 23 to 4 and 5, respectively), but it would be more expensive than strategy a (ICER of €61,763 and of €256,123 per additional case of DS detected, respectively) (see Fig. 6).

Given a rate of coverage of 89.97% for DS screening, strategy c would be more effective (detecting 341 DS cases instead of 271 with the current screening or 269 with NIPT as contingent testing and reducing the IT related miscarriages to 5 instead of 23 with the current screening) and more expensive than strategies a and b (ICER of €551,753 and €547,406 per additional case of DS detected, respectively).

For an analytic failure rate of NIPT of 0%, both for strategies b and c, the number of IT would decrease as compared to the base case (from 579 to 523 for strategy b and from 700 to 648 for strategy c), the number of invasive procedure-related miscarriages would remain the same and the number of DS cases detected would be the same for strategy b but would increase for strategy c (from 296 to 297). The total costs for both strategies would be lower than for the case base (for strategy b decrease from 8,111,351€ to 7,988,127€ and for strategy c from 41,395,745€ to 39,741,215€). Given a rate of analytic failure of the cffDNA tests of 12%, the number of IT conducted would increase as compared to the base case (from 579 to 853 for strategy b and from 700 to 964 for strategy c), the number of invasive procedure-related miscarriages would also increase (from 4 to 6 for strategy b and from 5 to 7 for strategy c) and the number of DS cases detected would be the same for strategy b and would decrease for strategy c (from 296 to 294). In this case, both strategies would be more costly than the base case (from 8,111,351€ to 8,512,953€ for strategy b and from 41,395,745€ to 45,165,330€ for strategy c).

Given a rate of coverage of 89.97% for DS screening and a cost of €150 per NIPT, strategy c would be more effective and more expensive than strategy b (ICER of €73,301 and €100,450 per additional case of DS detected, respectively). For the same coverage but at a cost per NIPT of €76, strategy c would be more effective and less expensive than strategy a, thus becoming the dominant alternative. In this scenario, strategy c would be more effective but more expensive than strategy b (ICER of €17,763 per additional case of DS detected).

## Discussion

Given that in Spain NIPT is mainly used in antenatal screening programmes by private healthcare providers, the objective of this study was to assess the potential impact of adding DNA-based test to the portfolio of services of the Spanish National Health Service. Based on data from the current ASP for DS in the Basque Country, we carried out a cost-effectiveness analysis comparing the first- and second-trimester screening strategies (current screening) to NIPT as contingent or as first-line screening strategy.

As reflected in the results of the base case analysed, NIPT as first-line testing detected more cases of DS and was associated with fewer miscarriages following invasive procedures, but with higher costs than the current screening. The use of NIPT as contingent testing detected fewer cases of DS (269 vs 271) but was associated with fewer miscarriages following invasive procedures (4 vs 23) and with lower costs, that is, it was less effective though also less expensive.

We consider that the deterministic sensitivity analysis conducted in our study is sufficient for the quantification of the uncertainty associated to the decision of adopting NIPT for the screening of DS. We have followed standard guidelines for cost-effectiveness research [30, 31]. As pointed out by the NICE guidelines, probabilistic sensitivity analyses are preferred when overall uncertainty needs to be characterised in the cost-effectiveness analysis due to the underlying uncertainty associated to all parameters included in the model [32]. Nevertheless, the main parameters used in our model were not subject to high levels of uncertainty, since they were real data obtained from the Basque Antenatal Screening Program (ASP) for Down syndrome and other chromosome anomalies, which provided all the information related to the current screening programme registered with the SsdwLab6© software and the 2015 List of Fees for Invoicing Healthcare and Teaching Services in the Basque Health Service (Osakidetza), which provided the real costs associated to the first- and second-trimester screening, invasive testing, karyotyping, pregnancy termination and hospitalization due to amniotic fluid leakage. Further, the sensitivity and specificity of NIPT were obtained from the reviews conducted by Benn et al. 2013 [25] and the Committee on Genetics Society for Maternal–Fetal Medicine in 2015 [26]. Namely, the sensibility and specificity values used for the model were 99.3% (95% CI 98.2% to 99.8%) and 99.84% (95% CI 99.69% to 99.92%), respectively. These values, are in agreement with those obtained in a more recent meta-analysis, in which pooled sensitivity and specificity were 99.3% (95% CI 98.9% to 99.6%) and 99.9% (99.9% to 100%), respectively for DS [10].

The sensitivity analysis indicated that results were sensitive to various different parameters: a reduction in the cost per NIPT; changes in the screening cut-off; screening uptake; analytic failure rate of NIPT and combined changes in the rate of coverage of prenatal screening and the cost per NIPT. A decrease of the cost of cffDNA tests to €150 or €76 would result in a reduction of the costs of the screening programme of 70.48% and 83.51%, respectively, using NIPT as primary testing and of 16.16% and 19.15%, respectively, using NIPT as contingent testing.

This implies that if the costs of DNA-based tests could be reduced to, for example, €76, the use of NIPT as first-line testing would be dominant when compared to the current screening. Such reductions in the cost per NIPT might be achieved either by exploiting the stronger negotiating position of public healthcare institutions compared to that of private laboratories offering these services, or by adopting NIPT specially designed to be implemented within the clinical laboratories of the Basque Health Service, using standard laboratory equipment and most of the current massive parallel sequencing systems. The latter option would result in cost savings through economies of scale, as well as a higher sample-processing capacity. Additionally, carrying out these tests using in-house resources would improve professional skills and standards in the existing genetic laboratories, with better quality control and shorter waiting times to obtain results.

Comparing with the current screening, the analysis indicates that the strategy of using NIPT as contingent testing would reduce the number of DS cases detected (271 vs 269), but would also decrease the number of procedure-related miscarriages from 23 to 4. In this regard, the scenario created if the screening cut-off was reduced to 1:500 or to 1:1000 should be given consideration. A reduction to 1:500 would lead to the detection of more cases of DS, with fewer invasive procedures being performed and fewer associated miscarriages, but at a higher cost (+ 3.5%) than the current screening (ICER of €61,763 per additional DS case detected). Depending on the price the healthcare system deems reasonable to pay to avoid a new case of DS, this strategy should be taken into account.

The scientific evidence suggesting that screening uptake in the general pregnant population would increase with the adoption of NIPT as compared to the current screening is still limited [33]. However, an increase of the screening uptake up to 89.97% for a screening programme in which NIPT would be used as first-line testing would detect more DS cases as compared to current screening (341 vs 271), would cause fewer number of invasive procedure-related miscarriages (5 vs 23), but at a much higher cost (47,524,553€ vs 8,901,872€).

The sensitivity analysis for the analytic failure rate of NIPT showed that when the failure rate was 0%, the number of IT would be reduced for both, NIPT as contingent testing and NIPT as first-line testing, the number of invasive procedure-related miscarriages would remain the same and the number of DS cases detected would be the same for NIPT as contingent testing and one more DS case would be detected if NIPT were used as first-line testing. When the NIPT failure rate was 12%, the number of IT tests would increase from 579 to 853 for NIPT as contingent testing and from 700 to 964 for NIPT as first-line testing, the number of invasive procedure-related miscarriages would increase by two cases for both strategies and the number of DS cases detected would remain the same when NIPT were used as contingent testing but would decrease by two cases when NIPT were used as first-line testing. The observed impact of the variation of the analytic failure rate on results is partly due to fact that the number of NIPT with positive results increases when the failure rate is 0% and decreases when the failure rate is 12%.

A key factor in the analysis is that the measurement of foetal nuchal fold (NF) thickness during the first trimester of pregnancy was included in all the strategies studied, since this is an important marker associated not only with DS but also with other chromosome anomalies and genetic disorders [26]. Several studies have confirmed that thanks to the measurement of NF thickness, with a screening cut-off within the 95% percentile (5% of false positives), the rate of T21 detection was 75%, rising to 80% if combined with maternal age [34]. It has also been observed that a thick NF can be associated with other chromosome anomalies including trisomy 18, trisomy 13, and Turner syndrome [35], as well as with foetal malformations, especially congenital heart defects and genetic syndromes [36] and that a combination of maternal age and NF thickness enables the calculation of the individual risk for a given pregnant woman. In order to achieve an accurate measurement of the NF, the performance of ultrasound scans requires a quality assurance system. Therefore, ultrasound scans should be performed by accredited sonographers in National Health Service centres.

This study was carried out for a genetic condition with a low prevalence (0.43%) in the general obstetric population. Hence, the positive predictive value of the screening tests, both current screening and NIPT, were very low given that few women with a positive result actually had an affected child. The sensitivity and specificity of the current prenatal screening programme tests for DS (based on data obtained from the ASP) were lower than those reported for NIPT (89.75% and 95.65% respectively vs 99.3% and 99.9%, respectively). Nonetheless, sensitivity and specificity values were higher than those found in other studies for first- and second-trimester screening (72.5% and 95% in the study conducted by Neyt et al. [37], 81% and 94.1% in the study carried out by Garfield and Armstrong [38], and a detection rate of 84% with a false positive rate of 4% in the study undertaken by Okun et al. [39]). The fact that in this study the number of cases of DS detected with the strategy based on using NIPT as first-line testing in the prenatal screening programme was only 9.2% higher than the number of cases detected using the current strategy, might be explained to a great extent by the high sensitivity and specificity of the screening tests of the ASP.

In the present study, NIPT was not considered diagnostic for the detection of T21. Hence, when NIPT results were positive women were offered confirmatory IT, as there is no substitute for the accuracy obtained by genetic analysis of chorionic villi or amniotic fluid samples. Additionally, the use of NIPT for the identification of other chromosome anomalies has certain limitations, which could potentially lead to false negatives [40]. Furthermore, cffDNA tests do not allow determining whether the trisomy is due to a translocation, which has an impact on the risk of recurrence [26].

In order to use NIPT as contingent testing in women with positive results it would be desirable to decrease the screening cut-off to 1:500 or 1:1000, since compared to the current first- and second-trimester screening it would improve the overall effectiveness of the programme increasing the number of DS cases detected (from 271 to 276 and 281, respectively) and decreasing the number of foetal losses (from 23 to 4 and 5, respectively), although the costs would be 3.5% higher. A recent study carried out in the United Kingdom, which evaluated the clinical implementation of NIPT as contingent testing following the results of the first-trimester combined test in routine clinical practice, estimated a reduction in invasive procedure rates of 43% [41]. The study indicated that the prenatal detection of trisomies and the result of the pregnancy would depend not only on the diagnostic accuracy of the screening tests, but also on the choice made by the pregnant women themselves. Hence, on the basis of the results of the contingent test in the high-risk group, 38% of women opted for IT, 60% for NIPT and 2% for not undergoing more follow-up tests; while in the moderate-risk group, 91.5% of women opted for NIPT and 8.5% for no follow-up tests. Therefore, in high-risk women, the adoption of NIPT occurred partially at the expense of IT, but mainly as a new option for women who would previously have chosen not to carry out more detection tests.

### Strengths and limitations of the study

The main strength of the study is that the economic analysis was developed on the basis of real data obtained from the Basque Antenatal Screening Programme for DS and other chromosome anomalies (ASP) and the results derived from the present cost-effectiveness analysis could be extrapolated to the Spanish National Health Service. Except for the cost per NIPT, which is highly variable in private clinical practice, all other costs were obtained from the 2015 List of fees for invoicing healthcare and teaching services in the Basque Health Service (Osakidetza) in accordance with the current legislation.

One limitation of the study is that despite NIPT can be used to test for trisomy 18, trisomy 13 and some sex chromosome aneuploidies, we have exclusively evaluated NIPT for the detection of DS. Nevertheless, trisomy in chromosomes 18 and 13 are most often lethal in utero or soon after birth and, therefore, detection of these aneuploidies would arguably pose limited benefit [42]. Besides, NIPT has a slightly lower sensitivity for trisomies 18 and 13 than for DS and in 100,000 pregnancies in the general obstetric population 154 and 42 false positive results would be expected for trisomies 18 and 13, respectively, given the low prevalence of these chromosome anomalies [10]. Another shortcoming of NIPT is that, unlike invasive screening tests, it does not detect other chromosomal rearrangements. Furthermore, only singleton pregnancies were considered, following the current recommendations of the American College of Obstetricians and Gynecologists [26]. In addition, the study assumed that the percentage of pregnant women with a positive NIPT who decide to terminate the pregnancy due to a diagnosis of T21 confirmed by invasive testing would be similar to that of the current screening. Nevertheless, the rate of invasive procedures carried out following a positive NIPT result might be higher, given that NIPT has a higher positive predictive value than the current screening [43].

Another limitation is the lack of data related to the uptake of NIPT under real world conditions. According to limited published evidence, the uptake of NIPT in the general pregnant population might be higher than for the current DS screening [33]. Nevertheless, real uptake values are not known. The economic analysis was performed assuming that the coverage for NIPT would be the same as for current DS screening, i.e. 78.38%. However, a bivariate sensitivity analysis was also conducted in which an increase of NIPT uptake of 89.97% was considered.

Finally, we should point out that the study had a short-time horizon (i.e., from week 10 of pregnancy until birth) and, therefore, long-term quality of life outcomes of people with DS were not considered. As a consequence, quality-adjusted life years (QUALYs) were not calculated. This fact could constitute a limitation for a genetic condition such as DS, for which if the affected foetus is born, there would be significant consequences in terms of costs and effects in the long term. We should also note that the effectiveness of the model was measured in accordance with an intermediate outcome measure (namely, the number of DS cases detected) and not with a final outcome measure (e.g., QUALYs).

## Conclusions

The use of NIPT as contingent testing in a screening programme for DS, based on a screening risk cut-off of ≥ 1:270 and at a cost of €550 per NIPT would detect fewer cases of T21 (269 vs 271 cases) but would decrease foetal losses due to IT (from 23 to 4). A decrease in the screening cut-off to 1:500 or 1:1000 would lead to an increase of the number of DS cases detected (276 and 281 cases detected, respectively) and would considerably decrease the number of foetal losses as compared to the current first- and second-trimester screening (from 23 to 4 and 5, respectively), with an extra cost of 3.5%.

The use of NIPT as first-line testing within a screening programme for DS, with each NIPT costing €550, seems to be beneficial compared to the current screening with a screening cut-off of ≥ 1:270, since it would increase the number cases of DS detected and would reduce the number of miscarriages following invasive procedures, but at much higher costs. Both, as first-line testing and as contingent testing when the screening cut-off is lowered ≥ 1:500, NIPT could become a dominant alternative to the current screening, if the price of the emerging DNA tests decreases to a level that is similar to that of current biochemical screening tests.

## Abbreviations

ASP: Basque Antenatal Screening Programme for Down syndrome and other chromosome anomalies
cffDNA: cell-free foetal DNA
CVS: chorionic villus sampling
DS: Down syndrome
ICER: incremental cost-effectiveness ratio
IT: invasive testing (i.e. amniocentesis and chorionic villus sampling)
NIPT: non-invasive prenatal testing
NF: nuchal fold
NT: nuchal translucency
T21: trisomy 21
